# Fluoroquinolone resistance among CTX-M producing uropathogenic *Escherichia coli* from HIV and non-HIV patients in South India

**DOI:** 10.1186/1471-2334-14-S3-P63

**Published:** 2014-05-27

**Authors:** Kesavaram Padmavathy, Padma Krishnan, S Rajasekaran

**Affiliations:** 1 Sree Balaji Dental College and Hospital, Bharath University, Chennai, India; 2 Dept of Microbiology, Dr. ALM PGIBMS, University of Madras, Chennai, India; 3 Government Hospital of Thoracic Medicine, Chennai, India

## Background

Extended spectrum β-lactamase (ESBL) especially, the CTX-M producing *Escherichia coli* has emerged world wide as the leading cause of community onset UTI in the era of antibiotic resistance. The CTX-M producers often exhibit co-resistance towards fluoroquinolones and are a growing challenge to patient care. The purpose of this study was to determine the incidence of fluoroquinolone resistance among CTX-M producing uropathogenic *E.coli* (UPEC).

## Methods

UPEC isolated from HIV (n=76) and non-HIV antenatal patients (n=42) were screened for ESBL production as per CLSI guidelines. *bla_CTX-M_* was detected by PCR. Susceptibility to ciprofloxacin was assessed as per CLSI guidelines. Fisher’s exact test (two tailed) was employed to analyze the statistical significance of the results.

## Results

ESBL producers were more common among the UPEC isolates from HIV compared to those from non-HIV patients (75% vs 52.4%, p=0.015, OR=2.7273). Significant difference was observed in the incidence of *bla_CTX-M_* among the ESBL producers from HIV and non-HIV patients (70.2% vs 31.8%, *p*=0.002, OR=5.042). Compared to the CTX-M non-producers, majority of the CTX-M producers were resistant to ciprofloxacin in both the groups (HIV, 92.5% Vs 58.8%, p=0.0037, OR=8.6333, and non-HIV, 71.4% vs 20%, p= 0.5235, OR=10).

## Conclusion

Fluoroquinolones are the most common non-β lactam antibiotic used in the treatment of infections caused by ESBL producing organisms. The results of our study suggest the possible emergence of plasmid mediated fluoroquinolone resistance among the CTX-M producing *E. coli*. The co-resistance exhibited by the CTX-M producers is a cause of concern, as it might facilitate the co-selection process.

